# The Role of Stroke on Verbal Fluency and Recall Trajectories: A Longitudinal Mixed-Effects Analysis Using ELSA

**DOI:** 10.1093/geroni/igaf122.4246

**Published:** 2025-12-31

**Authors:** Franklin Massa, Alejandra Marroig, Florencia Santiñaque, Graciela Muniz-Terrera

**Affiliations:** UDELAR, Montevideo, Montevideo, Uruguay; Universidad de la Republica Uruguay, Montevideo, Montevideo, Uruguay; UDELAR, Montevideo, Montevideo, Uruguay; Ohio University, Athens, Ohio, United States

## Abstract

Understanding cognitive decline is a challenge in aging populations, and stroke has emerged as a key event triggering both immediate and long-term impairments. Previous studies have shown that having a stroke is associated with acute impairment in cognitive domains. Furthermore, it may increase the risk of dementia and mortality. However, the evidence on how strokes model the trajectory of different cognitive domains is limited and inconsistent. We evaluated the association between having a stroke and trajectories of cognitive domains using 9 waves from the English Longitudinal Study of Aging (ELSA). We used the Verbal Fluency (VF) test to assess the executive function and Total Recall (TR) test for memory function. We adjusted linear mixed effects models to capture both within and between person variability using the time-to-stroke to model differences by age in cognitive trajectories. The results showed that having a stroke was associated with a decrease of 1.14 words (p < 0.001) in the VF mean performance at 70 years old. However, it was not associated with the rate of change of VF (p = 0.1206). For the TR, stroke corresponded to an average decrease of 0.66 words (p < 0.001) and a pronounced acceleration in the decline of 0.14 words on average per year (p < 0.001). These findings showed that stroke is associated with a significant immediate decrease in both cognitive domains. Additionally, memory function exhibits a significantly accelerated decline after stroke. These insights underscore the need for early cognitive monitoring and tailored interventions targeting memory-related functions following stroke.

